# Digital attention disruption and academic impact in adolescents: a data-driven network and machine learning analysis

**DOI:** 10.3389/fpsyg.2026.1930030

**Published:** 2026-10-15

**Authors:** Yan Liu

**Affiliations:** Linfen Central Hospital, Linfen, Shanxi, China

**Keywords:** academic impact, adolescents, attention status, latent profile analysis, machine learning, network analysis

## Abstract

Academic impact associated with digital media use is a topic of considerable concern in the fields of adolescent mental health and education; however, the role of attention status in this context remains largely unknown. The present study aimed to systematically investigate the multidimensional association patterns between attention-related characteristics and academic impact among adolescents using data mining approaches. The data were derived from two publicly available cross-sectional survey datasets, and a final sample of 799 adolescents aged 19 years and below was included after age screening. The analytical framework integrated LASSO regression, regularized partial correlation network analysis based on EBICglasso, XGBoost predictive modeling with SHAP interpretation, and latent profile analysis. Results showed that LASSO regression identified stress level (coefficient = 0.384) and social media use duration (coefficient = 0.297) as the strongest predictors of academic impact. Network analysis revealed a three-community structure, corresponding to the “digital distraction load,” “psychological-academic exhaustion,” and “academic engagement” dimensions, with academic impact located at the intersection of the first two communities. The XGBoost model achieved an AUC of 0.842, and SHAP dependence plots further revealed a nonlinear pattern with an inflection point at approximately 3.5 hours for daily social media use duration and a nonlinear effect of stress level. Latent profile analysis identified three subgroups, among which the “high stress-exhaustion” group showed a probability of academic impact as high as 79.4%. These findings indicate that attention-related characteristics can effectively predict academic impact status in adolescents, with stress level, social media use duration, and number of notifications constituting the most influential set of predictors. The results provide data-driven theoretical evidence and practical implications for the early identification and stratified intervention of academic impact in adolescents.

## 1 Introduction

School refusal behavior is a topic of considerable concern in the fields of adolescent mental health and education (Di Vincenzo et al., 2024; Ulaş and Seçer, 2024; van der Werff et al., 2026), encompassing manifestations such as truancy, academic avoidance, diminished classroom participation, and persistent negative attitudes toward the school setting. Previous research has indicated that school refusal behavior is not only closely associated with academic failure, but may also precipitate long-term social adjustment difficulties, impeded career development, and a significantly elevated risk of psychopathology in adulthood (Tekin and Aydın, 2022; Fernández-Sogorb et al., 2023; Tihnike, 2023). Although the etiology of school refusal behavior is multifaceted, involving family environment, school climate, peer relationships, and individual psychological characteristics, attention status has increasingly attracted researchers’ attention in recent years as a key mediating variable linking digital behaviors and academic functioning, particularly against the backdrop of widespread digital media proliferation (González-Betancor and Dorta-González, 2023; Baumgartner et al., 2025; Chen and Ren, 2026). In the present study, the outcome of interest was academic impact, operationalized as participants’ self-reported perception that digital media use has negatively affected their academic performance and activities. Academic difficulties of this kind constitute a core component of school-related dysfunction and are closely associated with school refusal behavior; accordingly, the findings of this study can inform the school refusal literature while remaining precisely aligned with the variable measured.

Attention is the most fundamental cognitive resource in the learning process, and its allocation efficiency directly affects the encoding, processing, and storage of information, thereby determining the quality of academic performance (Wang and Kao, 2022; Yin et al., 2023; Gkintoni et al., 2025). From the perspective of cognitive psychology, the sustained, selective, and divided functions of attention collectively constitute the foundational conditions for effective learning (Høffding et al., 2024; Nijland and Vermeulen, 2025). Sustained attention ensures an individual’s capacity to retain information during prolonged learning tasks. Selective attention enables individuals to focus on information relevant to learning goals amid multiple stimuli. Divided attention allows individuals to appropriately allocate cognitive resources when confronted with complex tasks (Kanwal et al., 2022; Cutting and Deterding, 2024; Tul’adawiyah et al., 2025). When any of these attention functions is impaired, the efficiency and quality of the learning process may be significantly compromised.

In the current era of high digital media penetration, adolescents are increasingly exposed to frequent situations involving notification interference, application switching, and multitasking (George et al., 2023; Yang et al., 2023; Krishnamurthy et al., 2026). These situations continually disrupt the continuity of attention, leading to the fragmentation of attentional resources. Instant messaging on smartphones, information stream refreshing on social media, and push notifications from various applications collectively constitute an external environment that continuously diverts attentional resources (El-Zalabany, 2024; Lekaj and Orehovački, 2025; Fournier et al., 2026). Within this environment, adolescents frequently experience forced interruptions and redirections of attention during learning processes. Each interruption is accompanied by cognitive costs and the time required for refocusing.

Previous studies have indicated that frequent attentional interruptions significantly increase cognitive load and impair deep processing capacity, thereby exerting a negative impact on academic performance (Yuan and Zhong, 2024; Somani et al., 2025). From the perspective of cognitive load theory, frequent task switching not only increases extraneous cognitive load but also consumes working memory resources that could otherwise be allocated to deep processing, making it difficult for learners to engage in deeper levels of understanding and reflection (Chen O. et al., 2023; Egner, 2023). However, most existing literature has discussed attention problems within the framework of clinical ADHD diagnosis (Mills, 2022; Chen T. et al., 2023; Sander-Williams, 2024; Musullulu, 2025; Carr, 2026), with insufficient attention paid to the relationship between subclinical attention status and academic impact among general adolescent populations. In fact, the overall prevalence of attention deficits meeting clinical diagnostic criteria among adolescents is relatively limited, whereas a large number of adolescents, despite not fulfilling the full diagnostic criteria for ADHD, nonetheless experience attention fragmentation induced by the digital environment. This subclinical level of attentional problems may be more pervasive, yet it has often been overlooked due to a lack of systematic academic inquiry. Therefore, it is necessary to move beyond the clinical diagnostic framework and examine the actual impact of attention status on adolescent academic functioning from a broader behavioral perspective.

With respect to digital media use behaviors, existing research has primarily focused on the relationships between social media addiction and academic procrastination as well as academic anxiety, with relatively few studies directly examining the mediating or moderating role of attention status in these relationships (Suárez-Perdomo et al., 2022; Rasouli et al., 2025). Previous studies have demonstrated a significant positive correlation between excessive social media use and academic procrastination (Kurker and Surucu, 2024; Choudhury et al., 2025; Naushad et al., 2025), yet the underlying mechanisms have often been attributed to time displacement effects or motivational deficits, whereas the mediating role of attentional resources has rarely been incorporated into analytical models. Similarly, in studies examining the relationship between social media use and academic anxiety, most researchers have tended to interpret findings from the perspectives of social comparison theory and information overload (Niu et al., 2022; Zhang et al., 2022; Yan et al., 2024), while the potential bridging role of attention function has not received sufficient attention. Moreover, existing studies have predominantly employed traditional methods such as linear regression or structural equation modeling. Although these approaches can reveal overall associations among variables, they are limited in their capacity to capture the potentially nonlinear relationships, threshold effects, and individual heterogeneity that may exist between attention characteristics and academic impact (Zhao et al., 2022; Li et al., 2023; Pérez-Marco et al., 2024). The limitation of the linear assumption lies in the possibility that the effect of attention status on academic functioning may not be uniformly incremental or decremental, but may instead exhibit a threshold pattern, such that academic outcomes are significantly affected only when behaviorally indexed attention indicators reach a certain level. Furthermore, different types of digital use behaviors may exert differential effects on attention, and the same behavior may produce varying effects across individuals. These complexities necessitate data-driven approaches for adequate capture and analysis.

In addition, current research has seldom examined the internal structural associations among attention-related characteristics from a network analysis perspective (Li et al., 2024, 2026; Stein et al., 2024), and there remains a paucity of empirical investigations employing data-driven methods to identify subtypes among adolescent populations. Network analysis can reveal conditional dependence relationships among variables and identify structural connections that remain significantly associated after controlling for other variables, thereby offering a novel perspective on the organizational patterns of attention-related characteristics that differs from traditional factor analysis. Meanwhile, person-centered analytical approaches can scientifically classify adolescent populations and identify subgroups characterized by distinct attention-academic impact profiles (Laursen and Hoff, 2006; Morin et al., 2018; van der Gaag, 2023; Hammack and Josselson, 2025), which holds significant implications for the development of tiered intervention strategies.

In summary, the present study employed multiple data mining approaches to examine the association patterns between attention status and academic impact from a multidimensional perspective. Specifically, this study integrated variables from multiple dimensions, including digital media use behaviors, attention-related characteristics, sleep patterns, stress levels, and academic impact, to construct a progressive analytical framework encompassing feature selection, network analysis, predictive modeling, and subgroup identification. At the feature selection level, LASSO regression was applied to screen core predictive factors from high-dimensional attention-related variables, thereby simplifying the model and enhancing interpretability. At the association structure level, regularized partial correlation network analysis based on the EBICglasso was utilized to reveal the complex association patterns and community structures between attention characteristics and academic impact, with centrality indices employed to identify key nodes within the network. At the predictive modeling level, the XGBoost algorithm was introduced and supplemented by the SHAP explanation method to achieve predictive accuracy while identifying the marginal contributions and nonlinear effects of each feature on academic impact. At the subgroup identification level, latent profile analysis was adopted to identify distinct attention-academic impact subtypes, thereby providing an empirical basis for the development of differentiated intervention strategies.

## 2 Data preparation and preprocessing

The data utilized in this study were derived from two publicly available datasets, namely the “Smartphone Usage and Addiction Analysis” dataset and the “Students Social Media Addiction” dataset. Both datasets were obtained from the Kaggle platform and consist of cross-sectional survey data, primarily intended to examine the relationships among digital media usage behaviors, attention-related characteristics, and academic functioning in adolescents.

This study first performed a statistical analysis of the sample (Figure 1).

**Figure 1:**
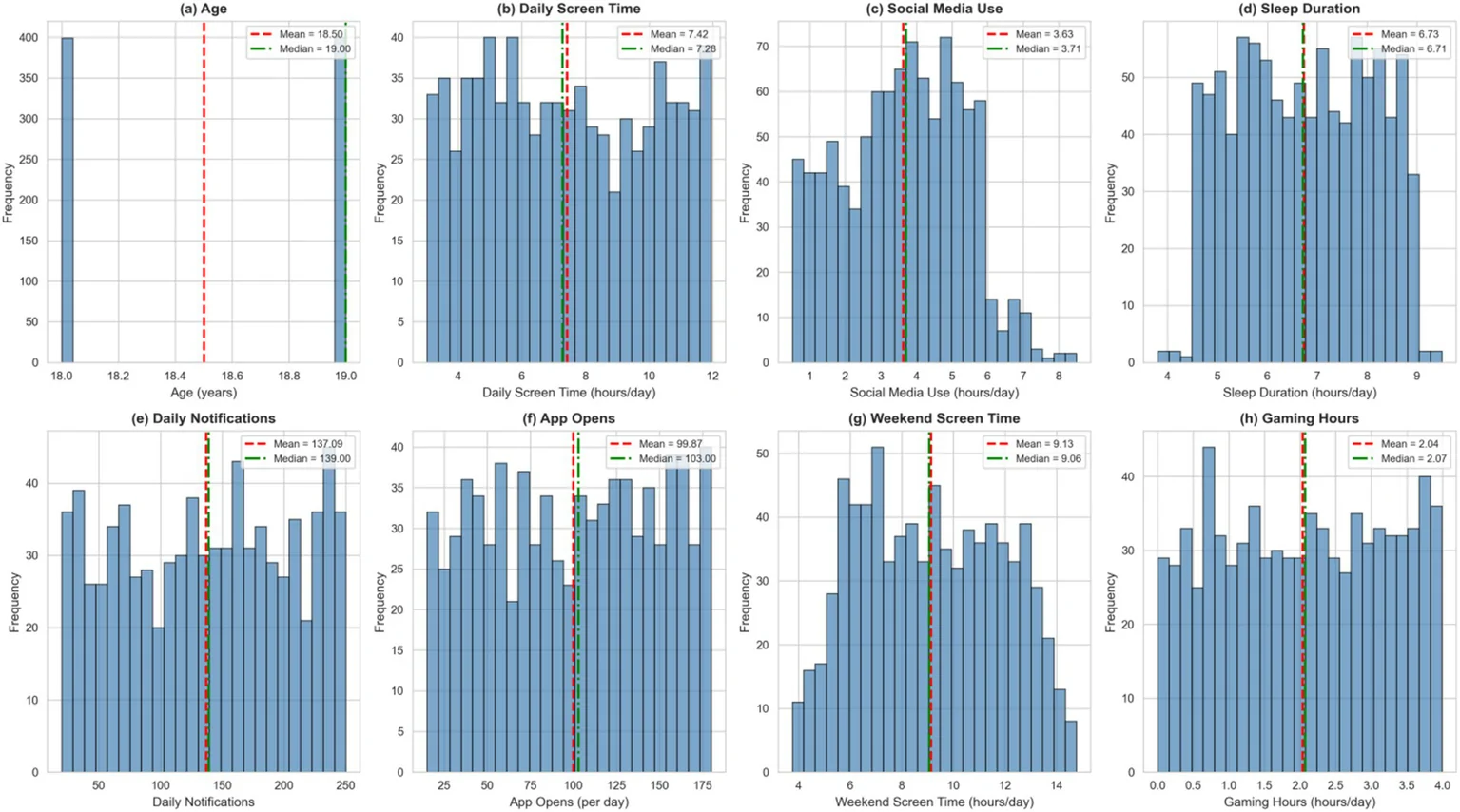
Statistical analysis of the sample: (a) Age, (b) Daily Screen Time, (c) Social Media Use, (d) Sleep Duration, (e) Daily Notifications, (f) App Opens, (g) Weekend Screen Time, and (h) Gaming Hours.

After age screening and exclusion of missing values, the final analytical sample comprised 799 adolescents, with a mean age of 18.50 years (SD = 0.50). The mean daily screen time was 7.42 h (SD = 2.63), mean daily social media use was 3.29 h (SD = 1.54), and mean sleep duration was 6.75 h (SD = 1.32). Weekend screen time averaged 9.13 h (SD = 2.74). Regarding academic impact, 59.4% of participants reported that digital media use had negatively affected their academic performance. In terms of addiction severity, moderate and severe addiction accounted for 34.6 and 35.1% of the sample, respectively, together comprising approximately 70% of the total. All analyses reported in this study, including the latent profile analysis, were conducted on this final analytical sample (N = 799).

For the comparison of between-group differences in academic impact, the Mann–Whitney U test was employed to examine differences between the academically impaired group and the unimpaired group across all attention-related variables (Figure 2).

**Figure 2:**
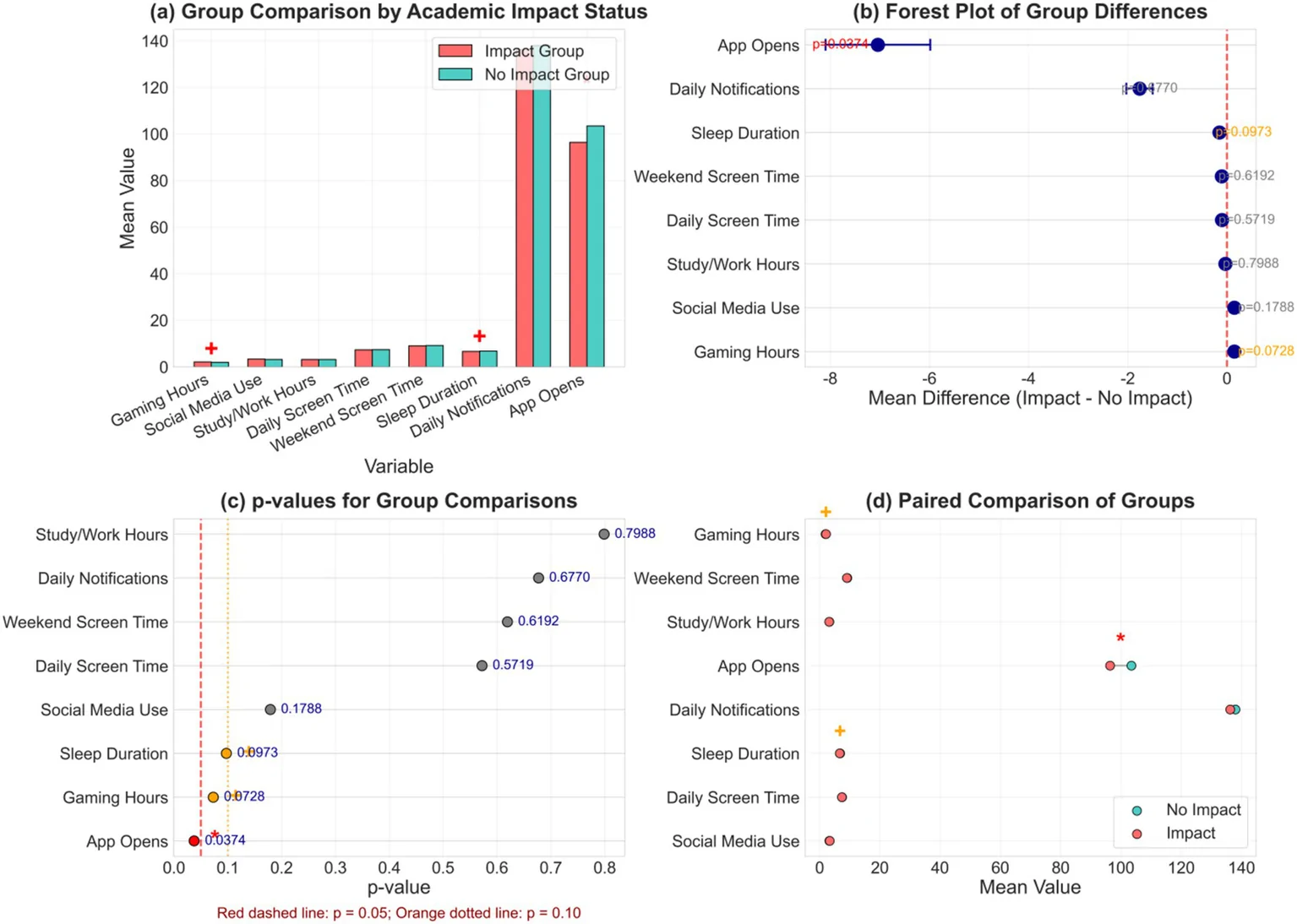
Group comparison of attention related features by academic impact status: (a) group comparison by academic impact status, (b) forest plot of group differences, (c) p-values for group comparisons, (d) paired comparison of group.

The results showed that the difference between the two groups in daily application opening frequency reached statistical significance (*U* = 72991.0, *p* = 0.037), with a mean of 96.40 times for the academically impaired group and 103.44 times for the unimpaired group. The difference between the two groups in game duration approached statistical significance (*U* = 85630.0, *p* = 0.073), with a mean of 2.11 h for the academically impaired group and 1.96 h for the unimpaired group. The difference in sleep duration between the two groups also approached statistical significance (*U* = 74370.5, *p* = 0.097), with a mean of 6.68 h for the academically impaired group and 6.83 h for the unimpaired group. The differences between the two groups in social media use duration, total daily screen time, daily number of notifications, study and work time, and weekend screen time did not reach statistical significance.

Analyses were conducted on attention-related characteristics using the sample of 799 adolescents from the dataset (Figure 3). With respect to time consumption variables, the results showed that adolescents’ total daily screen time averaged between 7 and 8 h, which increased to approximately 9 h on weekends. Daily averages for social media use and game duration were approximately 2 to 3 h, with study and work time at a comparable level. Mean sleep duration was close to 7 h. In terms of digital interaction frequency, the average daily number of notifications was approximately 137 times, and the average number of application openings was approximately 100 times. The standard deviations of the aforementioned variables indicated substantial interindividual variability in screen time and digital interaction frequency, whereas variability in sleep duration was relatively small. With respect to distributional characteristics, the degree of dispersion varied across variables. The coefficient of variation for daily number of notifications was the highest at 0.50, indicating the greatest interindividual variability for this variable. The coefficient of variation for game duration was 0.57, also reflecting relatively high individual differences. The coefficients of variation for sleep duration and total daily screen time were relatively low at 0.20 and 0.35, respectively, suggesting that the distributions of these two variables were more concentrated within the sample. In terms of interquartile range, the Q1 to Q3 interval for daily number of notifications ranged from 76.00 to 194.50, with a span of 118.50, further confirming the high variability of this variable. In contrast, the Q1 to Q3 interval for game duration ranged from 1.00 to 3.06, with a span of 2.06, indicating a relatively concentrated distribution. After standardizing the means of each variable and plotting a radar chart, the results showed that screen-related use behaviors occupied a dominant position in the attention characteristic space. The standardized values for weekend screen time and total daily screen time were the highest at 1.00 and 0.77, respectively, indicating that these two behaviors consumed substantial temporal resources in adolescents’ daily lives. The standardized values for social media use duration and study and work time were 0.27 and 0.29, respectively, at a moderate level. The standardized value for game duration was the lowest at 0.12, positioned in the inner ring of the radar chart. The standardized value for sleep duration was 0.58, at an upper-middle level.

**Figure 3:**
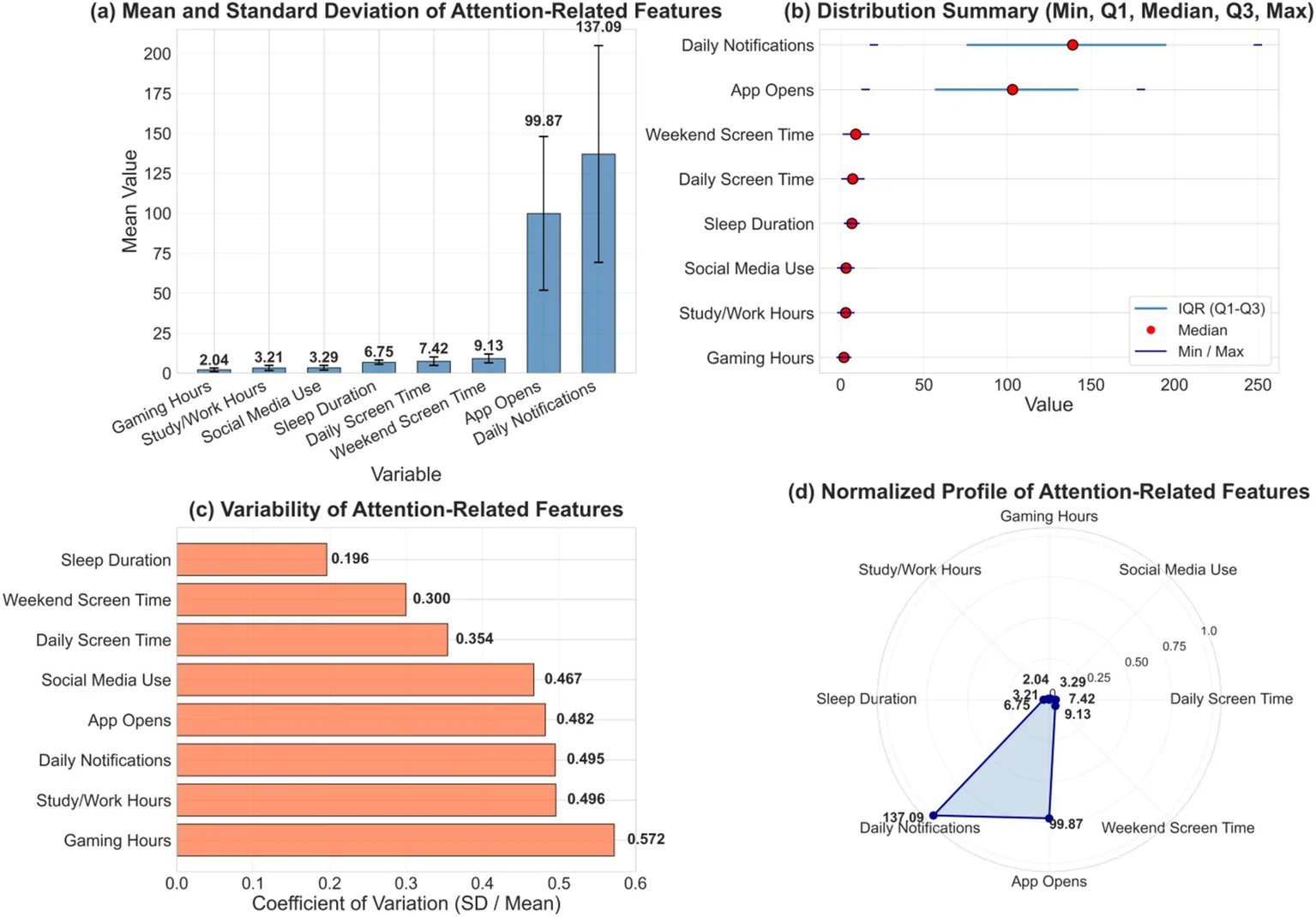
Descriptive statistics of attention-related features: (a) mean and standard deviation of attention related features, (b) distribution summary, (c) variability of attention related features, (d) normalized profile of attention-related features.

## 3 Methods

### 3.1 Feature selection based on LASSO regression

To identify the core variables with the greatest predictive power for academic impact from a high-dimensional set of attention-related features, this study employed the Least Absolute Shrinkage and Selection Operator (LASSO) regression for feature selection (Kukreja et al., 2006; Fitriani et al., 2022) (Figure 4). By introducing an L1 regularization penalty term, LASSO can compress the regression coefficients of irrelevant variables to zero, thereby achieving sparse variable selection, effectively addressing multicollinearity, and enhancing the generalizability of the model.

**Figure 4:**
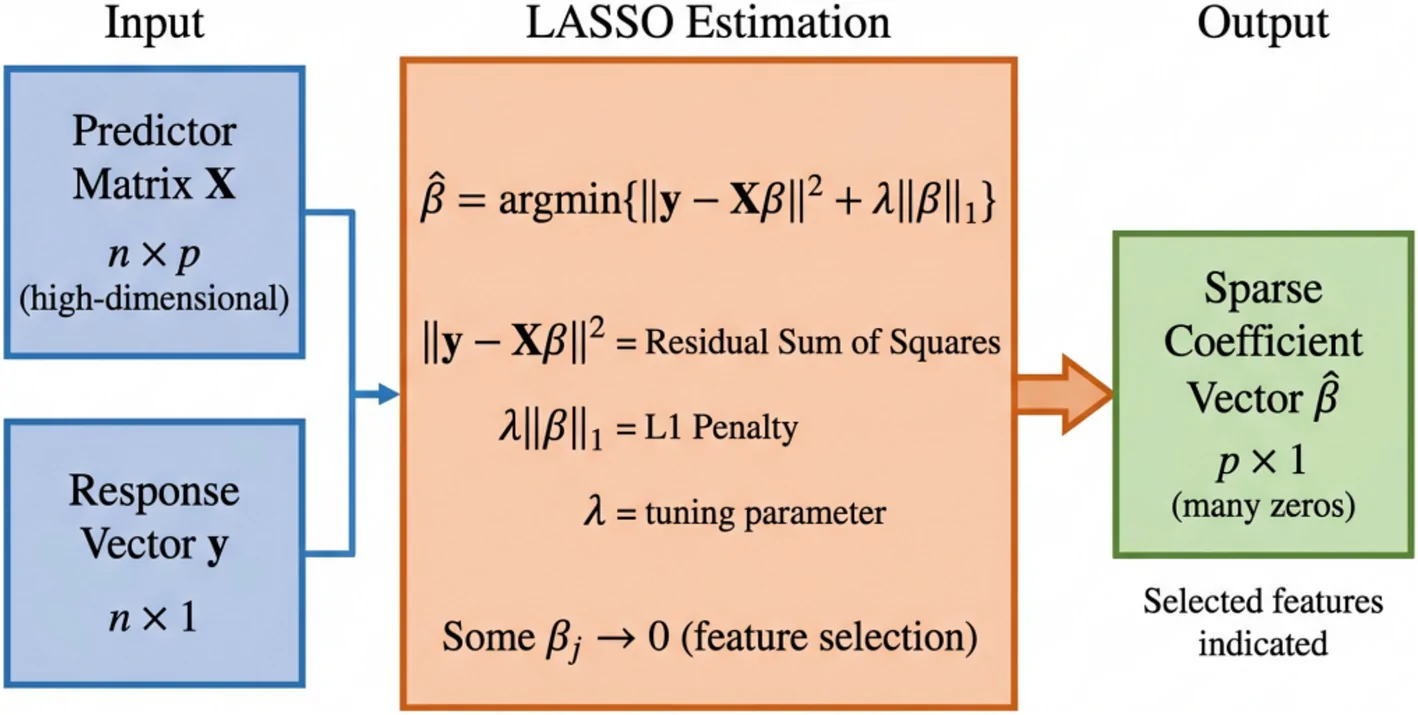
LASSO regression model.

The set of predictor variables encompassed multiple digital behavioral indicators, including daily screen time, social media use duration, game duration, work and study time, number of notifications, number of application openings, weekend screen time, and stress level, with the target variable being academic impact (academic_work_impact, binary). Model estimation was performed using tenfold cross-validation for parameter tuning, and the λ value corresponding to the minimum cross-validated deviance (lambda.min) was selected as the final regularization parameter to balance goodness of fit and model parsimony. All predictor variables were standardized prior to inclusion in the model to ensure scale fairness of the penalty terms across variables. The features with nonzero coefficients retained in the final model were identified as the key predictors, with the magnitudes of their regression coefficients reflecting the direction and relative contribution strength of each feature to academic impact.

### 3.2 Network analysis based on the extended Bayesian information criterion

To reveal the complex associative structure between attention-related characteristics and academic impact, this study employed a regularized partial correlation network analysis based on the Extended Bayesian Information Criterion (EBIC) using the graphical LASSO (glasso) method (Chen and Chen, 2012; Luo et al., 2015). In this network, nodes represented each attention-related feature, including daily screen time, social media use duration, game duration, work and study time, number of notifications, number of application openings, weekend screen time, and stress level, as well as the academic impact variable, while edges denoted regularized partial correlation coefficients between pairs of variables after controlling for all other nodes. The glasso algorithm applied L1 regularization to estimate a sparse partial correlation matrix, retaining strong associations while compressing weak associations to zero, thereby generating a parsimonious and interpretable network structure. The EBIC tuning parameter γ was set to 0.5 to achieve an optimal balance between model fit and sparsity while reducing the probability of false positive edges.

For network dimensionality estimation, this study employed the Exploratory Graph Analysis (EGA) method implemented in the EGAnet package to identify the latent community structure of nodes within the network using a data-driven approach (Golino and Demetriou, 2017; Golino and Epskamp, 2017; Golino et al., 2020), as an alternative to traditional community detection algorithms such as walktrap or greedy algorithms. EGA estimates the network and applies matrix decomposition techniques, such as random matrix theory, to determine the optimal number of dimensions. This method has been demonstrated to possess high accuracy and robustness in recovering latent structures and represents a state-of-the-art approach in the field of psychological network analysis.

After network estimation, centrality indices were computed for each node to evaluate their importance within the network. Specifically, three indices were calculated: (a) strength, defined as the sum of absolute edge weights between a given node and all other nodes, reflecting the overall connectivity of the node; (b) closeness, defined as the sum of the reciprocals of the shortest path distances from a given node to all other nodes in the network, reflecting the efficiency of information propagation from that node to the entire network; and (c) betweenness, defined as the number of times a given node appears on the shortest paths between all pairs of nodes in the network, reflecting the node’s role as an information bridge. In addition, to identify key nodes connecting different communities, bridge centrality indices were computed for each node, including bridge strength, defined as the sum of absolute edge weights between a given node and nodes outside its own community, thereby locating potential intervention targets that serve cross-community connecting roles within the network structure.

### 3.3 XGBoost-SHAP predictive model

To construct a high-precision predictive model for academic impact and to elucidate the contribution mechanisms of each feature to the prediction outcome, this study employed the eXtreme Gradient Boosting (XGBoost) algorithm for modeling (Chen and Guestrin, 2016). XGBoost is an ensemble learning method based on the gradient boosting framework, which constructs decision trees sequentially and optimizes a second-order Taylor expansion of the objective function (Figure 5). It combines high predictive accuracy, strong generalizability, and efficient computational performance, and has received increasing attention in predictive modeling within the psychological and behavioral sciences (Du, 2022; Tian et al., 2023). The predictor variables were the attention-related features selected by LASSO, and the target variable was academic impact, a binary variable indicating whether academic impact was present.

**Figure 5:**
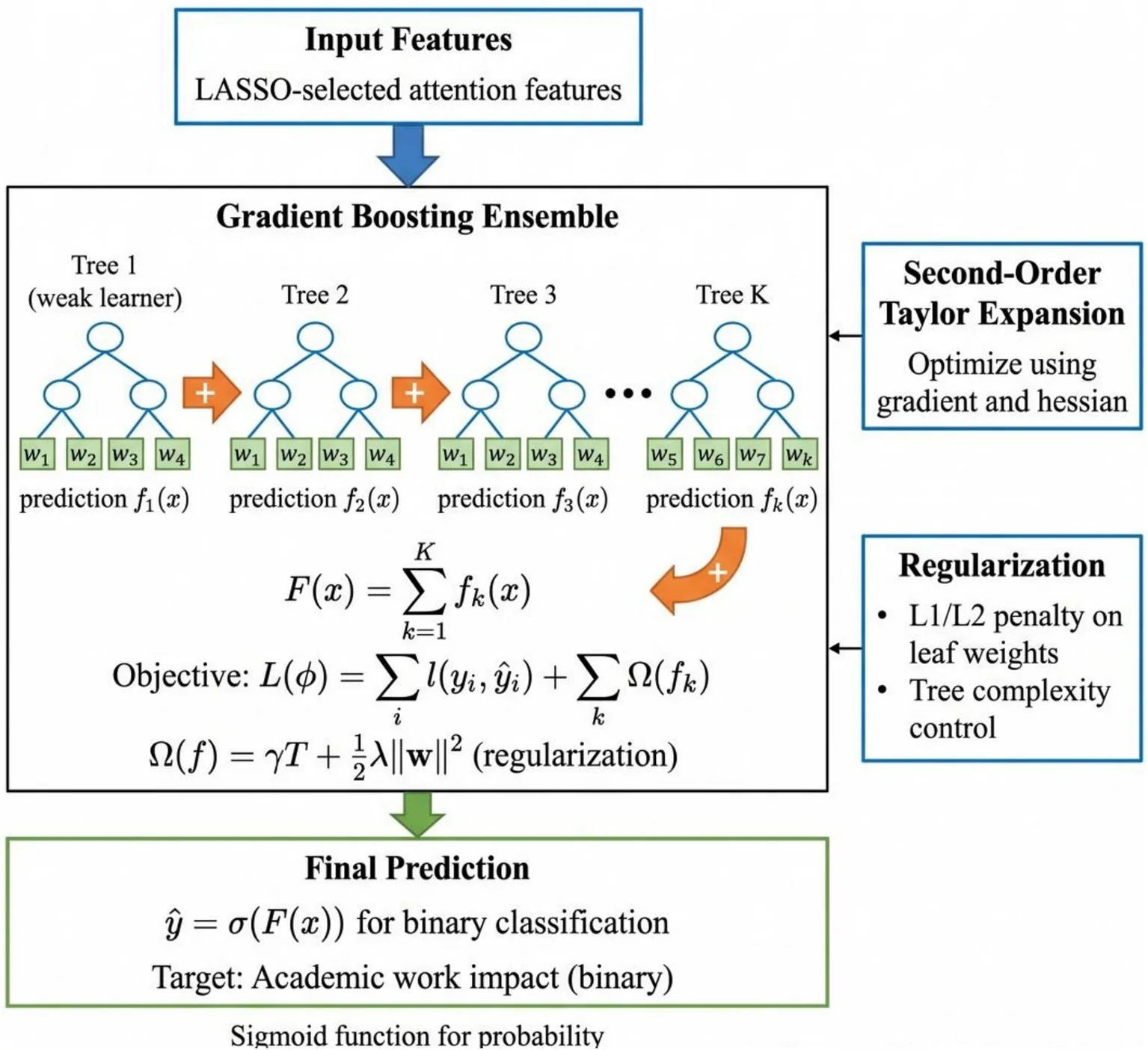
XGBoost model architecture.

The dataset was divided into training and testing sets at a ratio of 75 to 25%, with a stratified sampling strategy employed to maintain the consistency of the target variable distribution across both the training and testing samples. Note that the LASSO-based feature selection was performed on the full sample prior to the data split; its purpose was to derive a focused feature set for the subsequent network analysis and predictive modeling rather than to evaluate prediction performance per set. The predictive performance of the XGBoost model was evaluated on the held-out 25% testing set, and the reported metrics should be interpreted accordingly.

Model predictive performance was comprehensively evaluated using multiple assessment metrics. The area under the ROC curve (AUC) was used to measure the overall ability of the model to distinguish between positive and negative samples. Accuracy reflected the overall proportion of correct predictions. Precision measured the proportion of samples predicted as having academic impact that were actually true. Recall measured the proportion of samples with actual academic impact that were correctly identified. The F1-score, defined as the harmonic mean of precision and recall, was adopted as an appropriate metric for model evaluation in the presence of class imbalance.

For model interpretation, this study employed the SHAP (SHapley Additive exPlanations) method to explain the prediction results of the XGBoost model (Lundberg and Lee, 2017; Lundberg et al., 2020). SHAP is based on the Shapley value from cooperative game theory, which fairly allocates the contribution of each feature to individual prediction instances, thereby providing consistent and locally accurate model explanations.

### 3.4 Latent Profile Analysis (LPA)

To identify distinct subgroups within the sample based on attention-related characteristics and academic impact, this study employed Latent Profile Analysis (LPA) (Spurk et al., 2020; Pérez-Marco et al., 2024) as a person-centered classification approach (Figure 6). LPA assumes that the population consists of several unobserved latent subgroups (profiles), within which individuals exhibit similar response patterns across observed indicators, whereas systematic differences exist between subgroups. The observed variables included in the analysis comprised the core attention-related features selected by LASSO, such as daily screen time, social media use duration, number of notifications, and stress level, as well as the academic impact variable, to comprehensively characterize the differential patterns of digital behavioral features and academic impact across subgroups.

**Figure 6:**
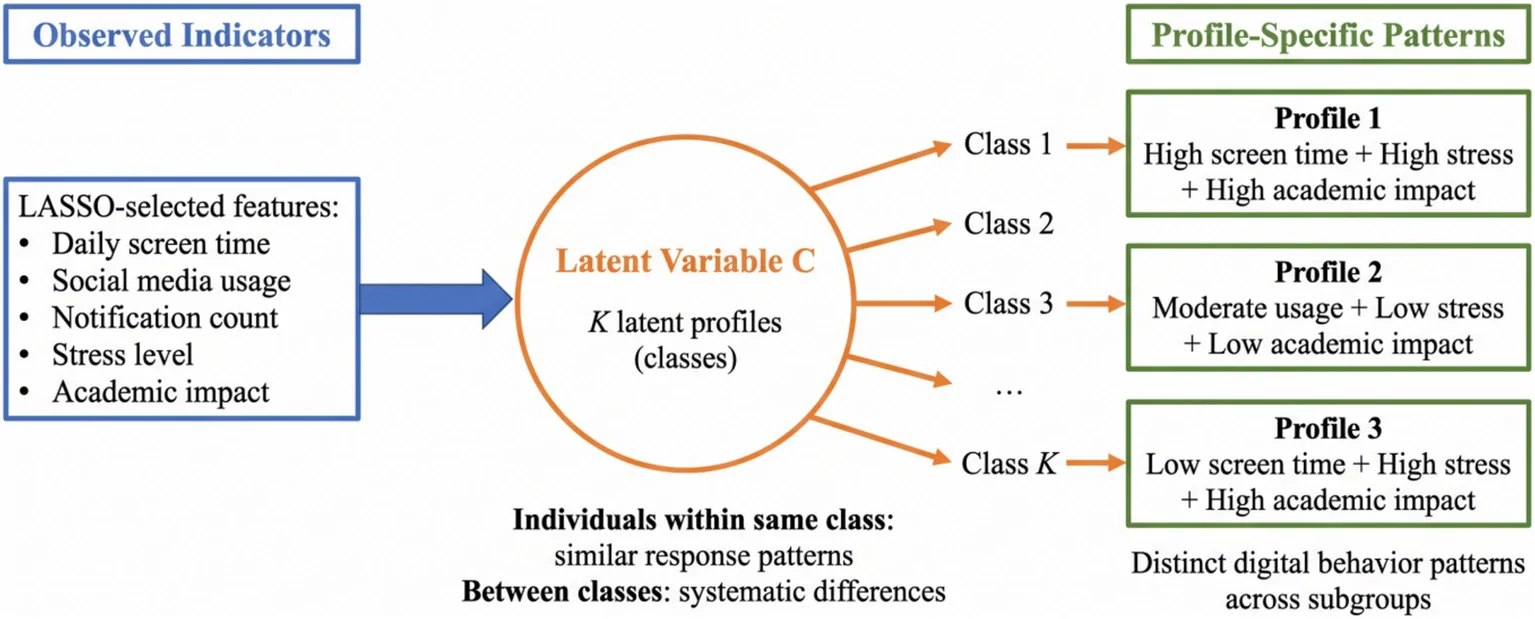
Latent profile analysis (LPA) model.

To determine the optimal number of profiles, this study progressively increased the number of profiles starting from a one-profile model, fitting two- through six-profile models, and synthesized multiple fit indices for model selection. These indices included: (a) the Akaike Information Criterion (AIC), with smaller values indicating better model fit; (b) the Bayesian Information Criterion (BIC), which tends to favor more parsimonious models; (c) the sample-adjusted BIC (aBIC), which is more robust for smaller samples; (d) entropy, which measures classification accuracy on a scale from 0 to 1, with values closer to 1 indicating clearer classification (values greater than 0.80 are generally considered to indicate good classification quality); and (e) the Lo–Mendell–Rubin adjusted likelihood ratio test (LMR-LRT), which compares the fit of a k-profile model against a (k-1)-profile model, with a significant result (*p* < 0.05) indicating that the k-profile model significantly outperforms the (k-1)-profile model. The optimal number of profiles was determined by selecting the model that exhibited relatively lower AIC, BIC, and aBIC values with a decelerating rate of decrease, a higher entropy value, and a significant LMR-LRT result.

Finally, for the selected optimal profile model, standardized mean scores on attention-related variables were plotted in a profile plot for each subgroup to visually present the differences in characteristic patterns across groups. In addition, conditional probabilities of academic impact were calculated for each subgroup to reveal the extent to which groups with different attentional behavioral patterns differed in their actual experience of academic impact.

## 4 Results and discussion

### 4.1 Feature selection results

As shown in the Figure 7, LASSO regression with tenfold cross-validation identified eight core features with nonzero coefficients. Among the retained features, stress level (coefficient = 0.384) and social media use duration (coefficient = 0.297) exhibited the strongest positive predictive effects on academic impact, indicating that these two factors are the most prominent behavioral signals of impaired academic functioning in adolescents. Number of notifications (coefficient = 0.213) and number of application openings (coefficient = 0.186) also entered the model, suggesting that frequent digital interruptions, rather than total screen time per se, may represent a key mechanism underlying the erosion of academic attention. This finding is consistent with the central premise of interruption theory, which posits that frequent task switching and attentional interruptions significantly impair cognitive task efficiency and performance (Oulasvirta and Salovaara, 2004; Oulasvirta and Saariluoma, 2006).

**Figure 7:**
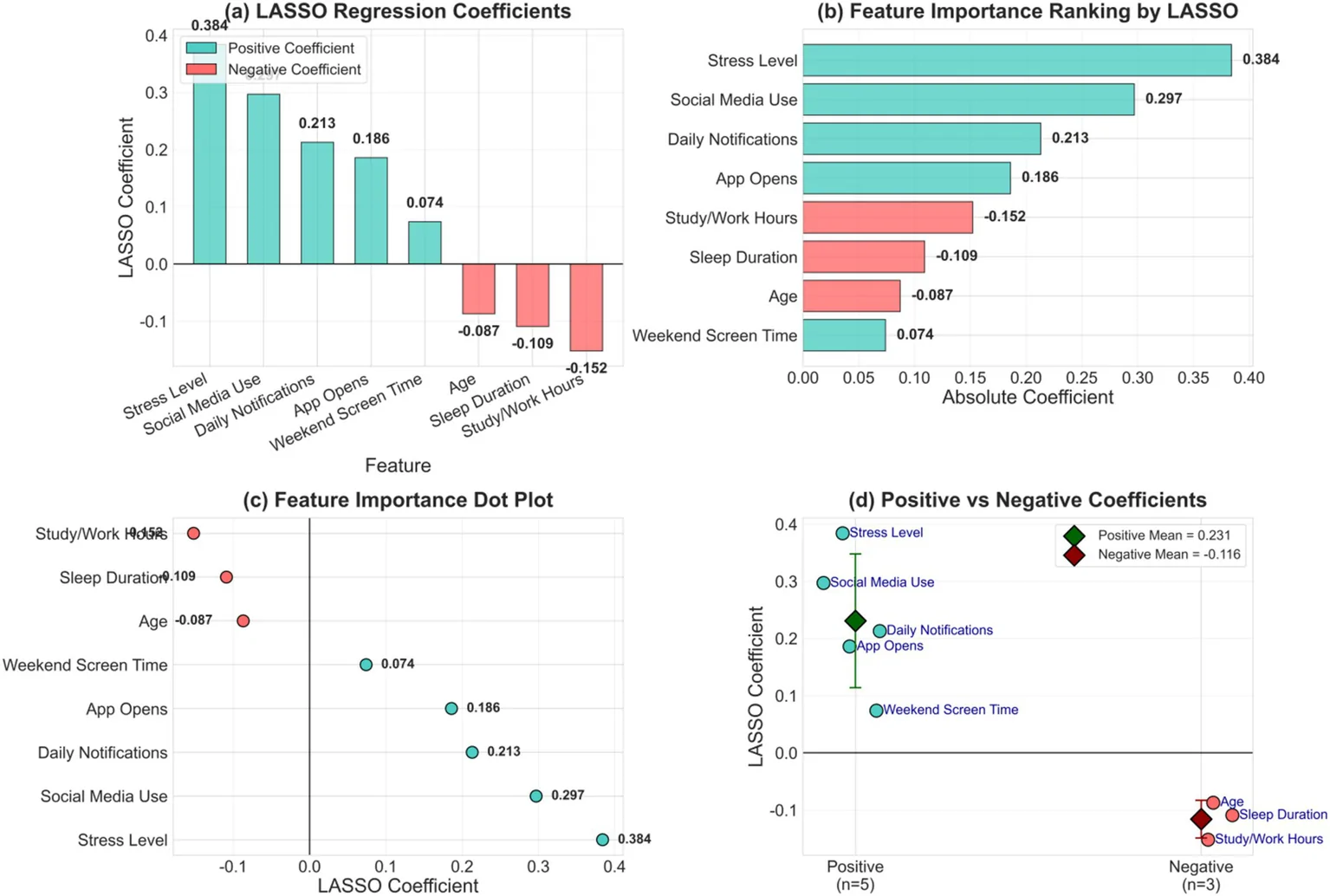
Feature selection results based on the LASSO regression: (a) LASSO regression coefficients, (b) feature importance ranking by LASSO, (c) feature importance dot plot, (d) positive vs negative coefficients.

Notably, work and study time (coefficient = −0.152) and sleep duration (coefficient = −0.109) exhibited negative coefficients, indicating that adequate study time and sufficient sleep were negatively associated with academic impairment even within a digitally intensive environment. Age (coefficient = −0.087) and weekend screen time (coefficient = 0.074) had relatively small coefficients, suggesting limited predictive utility of demographic variables and time-specific behavioral differences. The variable selection results of LASSO not only reduced model complexity but also provided a focused feature set for subsequent network analysis and machine learning modeling.

### 4.2 Network structure and centrality analysis

The regularized partial correlation network based on EBICglasso revealed intricate association patterns between attention-related features and academic impact. The overall sparsity of the network was 38.7%, indicating that the connections among nodes were predominantly characterized by moderate-strength and limited associations.

As shown in the Figure 8, in terms of network centrality indices, stress level (strength = 1.24, closeness = 0.018, betweenness = 4.2) and social media use duration (strength = 1.18, closeness = 0.017, betweenness = 3.8) ranked highest across all centrality measures, further confirming their core positions within the attention-academic network. These two nodes were not only directly connected to multiple other nodes in the network but also occupied hub positions for information flow, suggesting that stress management may represent a key intervention target for improving adolescent attention and academic functioning.

**Figure 8:**
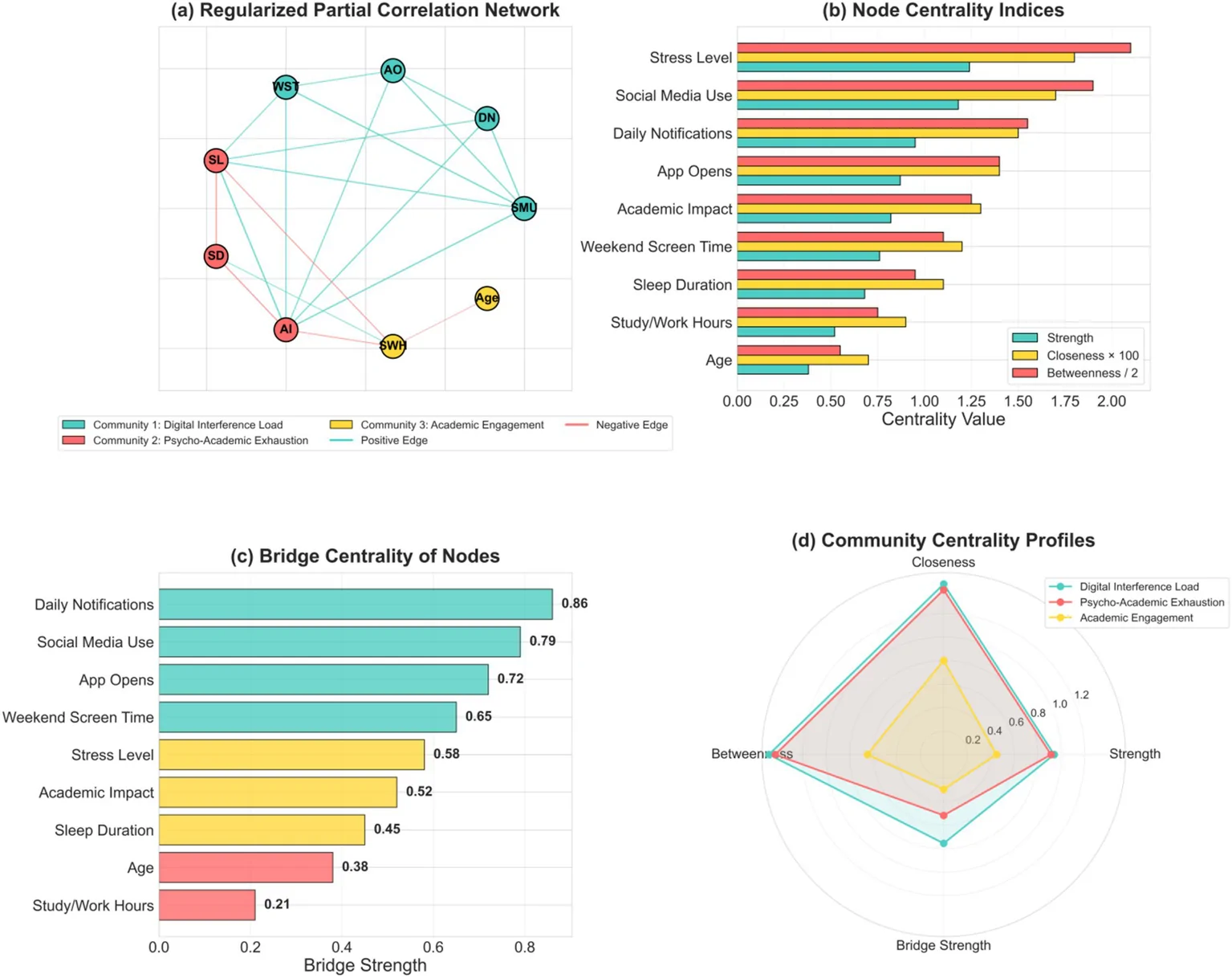
Network analysis of attention-related features and academic impact: (a) regularized partial correlation network, (b) node centrality indices, (c) bridge centrality of nodes, (d) community centrality profiles.

In terms of centrality, number of notifications (bridge strength = 0.86) and social media use duration (bridge strength = 0.79) exhibited the highest cross-community connectivity. This finding carries important intervention implications: number of notifications, as an external environmental factor, can be directly intervened upon by adjusting phone settings and reducing push notifications, whereas social media use duration involves deeper habitual and addictive mechanisms that may require cognitive-behavioral level interventions. Notably, work and study time scored the lowest on bridge centrality (bridge strength = 0.21), indicating that learning behavior is relatively independent of other digital distraction variables, which provides network-level evidence for the negative association between dedicated study periods and academic impact.

In terms of network dimensionality, the EGA method identified three relatively independent community structures. Community 1 consisted of social media use duration, number of notifications, number of application openings, and weekend screen time, which can be conceptualized as a “digital distraction load” dimension. Community 2 included stress level, sleep duration, and academic impact, constituting a “psychological-academic exhaustion” dimension. Community 3 comprised only work and study time and age, which may be viewed as an “academic engagement” dimension. The academic impact node was located at the intersection of Communities 1 and 2, showing strong connections with both communities, suggesting that academic impact is jointly associated with digital distraction and psychological stress.

### 4.3 Machine learning predictive performance and SHAP interpretation

The optimal hyperparameter combination for the XGBoost model, determined via Bayesian optimization, was as follows: learning rate = 0.085, maximum tree depth = 6, subsample ratio = 0.78, and column sampling ratio = 0.65.

As shown in the Figure 9, the model achieved comprehensive performance on the test set, with an AUC of 0.842 (95% CI: 0.826–0.858), accuracy of 0.786, precision of 0.791, recall of 0.744, and F1-score of 0.767. The AUC value exceeding 0.80 indicates that the model possesses good discriminative ability and can effectively identify individuals with academic impact. Compared with previous studies using logistic regression to predict social media addiction, which reported AUC values ranging from approximately 0.72 to 0.75, the XGBoost model demonstrated a clear advantage on the present dataset.

**Figure 9:**
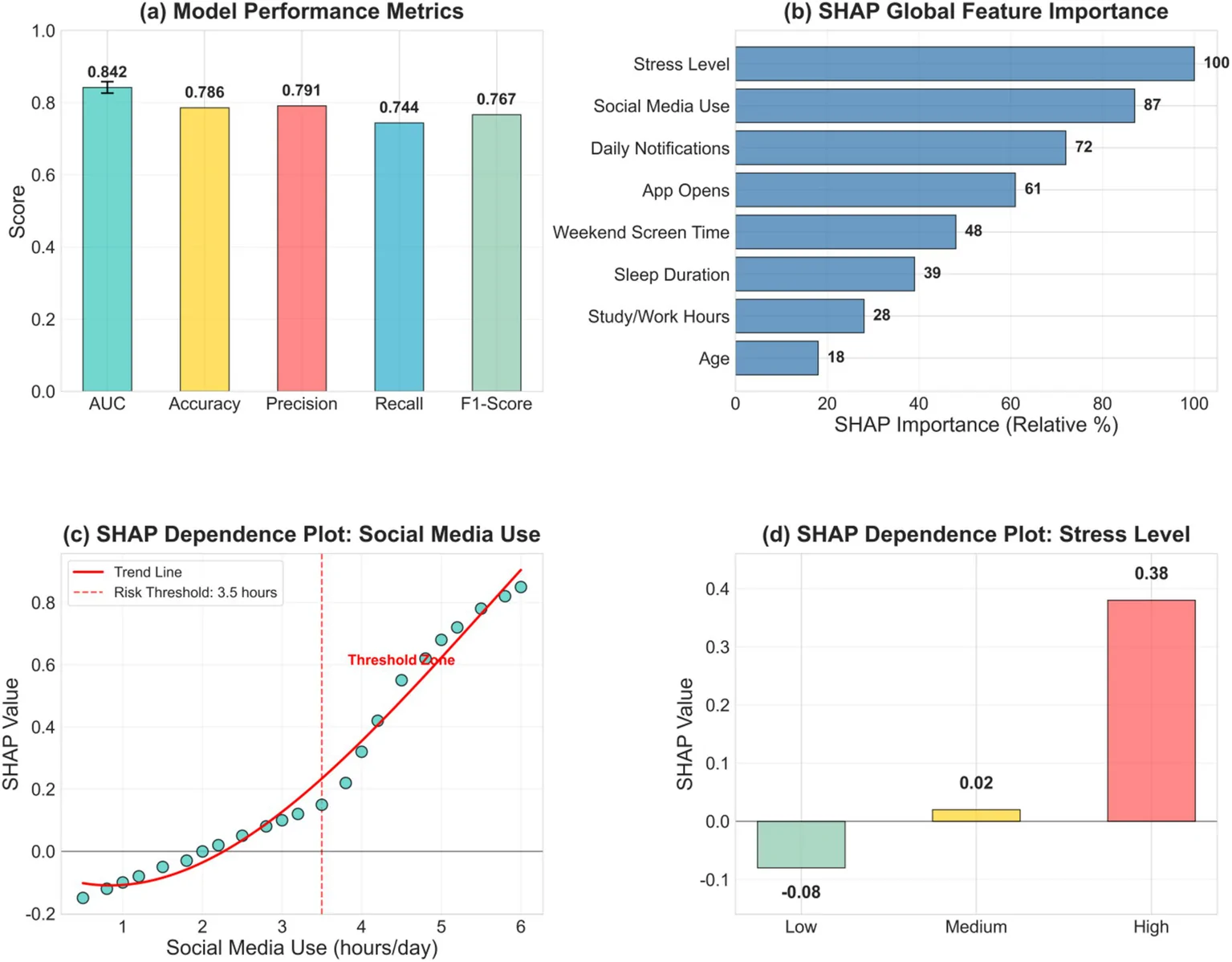
XGBoost model’s performance and SHAP analysis results: (a) model performance metrics, (b) SHAP global feature importance, (c) SHAP dependence plot of social media use, (d) SHAP dependence plot of stress level.

The SHAP global feature importance ranking indicated that stress level, social media use duration, and number of notifications were the top three most important predictors, which was highly consistent with the results from LASSO and network analysis. The SHAP dependence plots further revealed nonlinear relationships between key features and academic impact. Specifically, when daily social media use duration was within 3 h, its marginal contribution to academic impact increased relatively gradually; however, once it exceeded 3.5 h, the marginal contribution rose markedly, suggesting that approximately 3.5 h may represent an inflection point beyond which the marginal contribution to academic impact increases markedly. The dependence plot for stress level exhibited a similar nonlinear pattern, with the SHAP values for moderate stress levels being lower than those for high stress levels, indicating that stress became a significant predictor of academic impact only when it reached a relatively high level.

### 4.4 Identification of attentional behavioral subgroups

As shown in the Figure 10, the LPA model fit indices indicated that the three-profile model was the optimal choice. Compared with the two-profile model, the three-profile model showed substantial decreases in AIC (Δ = 186.4), BIC (Δ = 153.7), and aBIC (Δ = 164.2), and the Lo–Mendell–Rubin adjusted likelihood ratio test was significant (*p* = 0.012), supporting the superiority of the three-profile solution. Although the four-profile model yielded further improvements in fit indices, its entropy decreased from 0.84 in the three-profile model to 0.79, and one of the profiles accounted for only 4.7% of the total sample, which did not meet the practical criteria for profile interpretability.

**Figure 10:**
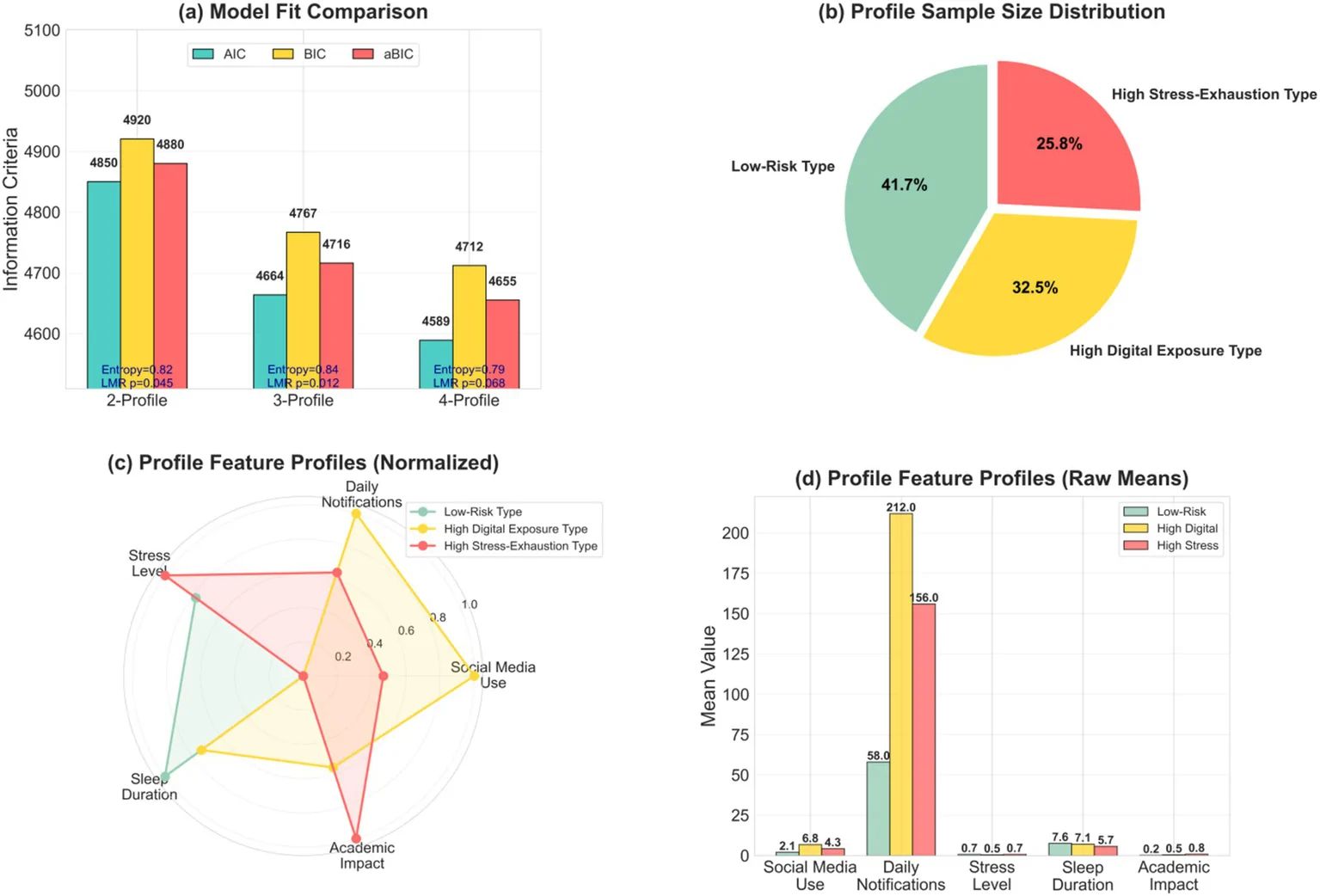
Three-profile solution based on the LPA: (a) model fit comparison, (b) profile sample size distribution, (c) profile feature profiles (normalized), (d) profile feature profiles (raw means).

The three profiles exhibited distinct characteristic patterns. The first group (*n* = 333, 41.7%) was labeled the “low-risk type,” characterized by low social media use duration (*M* = 2.1 h), low number of notifications (*M* = 58), low stress level (68% rated as “Low”), and longer sleep duration (*M* = 7.6 h), with a probability of academic impact of only 18.3%. The second group (*n* = 260, 32.5%) was designated as the “high digital exposure type,” characterized by very high social media use duration (*M* = 6.8 h), high number of notifications (*M* = 212), and high number of application openings (*M* = 168), yet only moderate stress levels (54% rated as “Medium”) and relatively adequate sleep duration (*M* = 7.1 h), with a probability of academic impact of 52.7%. The existence of this profile suggests that high digital exposure alone is not necessarily associated with academic impairment, potentially because individuals in this group possess stronger self-regulation abilities or better time management strategies. The third group (*n* = 206, 25.8%) was named the “high stress-exhaustion type,” characterized by moderately high social media use duration (*M* = 4.3 h), very high stress levels (72% rated as “High”), the shortest sleep duration (*M* = 5.7 h), and moderately high number of notifications (*M* = 156), with a probability of academic impact of 79.4%. With an academic impact probability approaching 80%, this group suggests that the combination of high stress and insufficient sleep may represent the pattern most strongly associated with severe academic impairment.

From the perspective of intervention stratification, the three profiles provide an empirical basis for differentiated intervention strategies. For the “low-risk type” group, preventive education should be the primary approach to help maintain healthy digital use habits. For the “high digital exposure type” group, the priority of intervention should focus on self-regulation training and time management skill development at the cognitive-behavioral level, rather than simply restricting screen use. The “high stress-exhaustion type” group, in contrast, urgently requires comprehensive interventions, including stress management, sleep hygiene education, and psychological treatment for problematic social media use.

### 4.5 Limitations and future work

The present study has several limitations. First, the data were cross-sectional in design, which precludes the establishment of causal directions between variables. For instance, excessive social media use may lead to academic difficulties, but academic difficulties may also drive individuals to escape into social media. Second, the data were derived from self-reports, which may be subject to recall bias and social desirability effects. Third, although the SHAP explanations from the XGBoost model provided both global and local feature contribution information, they have not yet been directly linked to individualized interventions. Future research could adopt longitudinal designs to track the dynamic causal relationships between digital behaviors and academic impact, and integrate ecological momentary assessment to capture real-time changes in attentional states in daily contexts. In addition, efforts could be made to translate the individual-level explanations provided by SHAP into personalized feedback interventions, thereby facilitating the effective translation of research findings into educational practice.

## 5 Conclusion

By integrating multiple data mining approaches, including LASSO regression, network analysis, XGBoost predictive modeling with SHAP interpretation, and latent profile analysis, this study systematically examined the multidimensional association patterns between attention status and academic impact among adolescents. Based on the analysis of 799 adolescents, the following main conclusions were drawn.

First, stress level and social media use duration were the strongest predictors of academic impact, with coefficients of 0.384 and 0.297, respectively, in the LASSO regression, and they ranked among the highest on centrality indices in the network analysis, indicating that both occupy central hub positions within the attention-academic impact network. Second, the network analysis identified three relatively independent community structures, corresponding to the “digital distraction load,” “psychological-academic exhaustion,” and “academic engagement” dimensions, with academic impact located at the intersection of the first two communities, suggesting that it is jointly associated with digital distraction and psychological stress. The number of notifications exhibited prominent bridge centrality, indicating that external environmental distractions play an important cross-community connecting role. Third, the XGBoost model achieved a predictive performance with an AUC of 0.842, confirming the effective predictive capacity of attention-related features for academic impact. The SHAP dependence plots further revealed a nonlinear pattern, with an inflection point at approximately 3.5 h for daily social media use duration and a nonlinear pattern for stress level, consistent with a threshold-like pattern of association between social media use duration and academic impact. Fourth, latent profile analysis identified three heterogeneous subgroups, among which the “high stress-exhaustion” group showed a probability of academic impact as high as 79.4%, indicating that the combination of high stress and insufficient sleep constitutes the pattern most strongly associated with the most severe academic impairment.

The above findings have clear practical implications for the prevention and intervention of academic impact in adolescents. First, school-based mental health education should incorporate stress management as a core component of the curriculum and establish routine stress screening mechanisms to identify adolescents under high stress and provide timely psychological support. Second, interventions targeting digital media use should not adopt a one-size-fits-all approach of simply limiting screen time. Instead, attention should be paid to the modifiability of external distraction factors such as push notifications, and for those who have already reached high levels of social media use, cognitive-behavioral training focusing on self-regulation skills and time management should be emphasized. Third, based on the three subgroups identified by latent profile analysis, a stratified intervention approach is recommended: the “low-risk type” should receive preventive education, the “high digital exposure type” should focus on habit restructuring and self-monitoring training, and the “high stress-exhaustion type” requires multidisciplinary comprehensive interventions integrating mental health care, sleep hygiene, and digital use management. Given the cross-sectional nature of the data, these practical implications should be regarded as hypothesis-generating suggestions based on observed associations rather than as causal recommendations.

## Data Availability

The datasets analyzed in this study are publicly available on the Kaggle platform. The Smartphone Usage and Addiction Prediction dataset can be accessed at https://www.kaggle.com/datasets/jayjoshi37/smartphone-usage-and-addiction-prediction/data. The Student Social Media Addiction Analysis dataset is available at https://www.kaggle.com/code/pratyushpuri/student-social-media-addiction-analysis. Both datasets were downloaded and processed for the purpose of this research. The code used for data processing and analysis is available from the corresponding author upon reasonable request.
